# Gonadal development in scorpion mud‐turtles, *Kinosternon scorpioides*, in a controlled environment

**DOI:** 10.1002/ar.70116

**Published:** 2025-12-24

**Authors:** Brenda Braga, Verônica Oliveira‐Bahia, Maria Auxiliadora Ferreira, José Ribamar Felipe Marques, Diva Araújo Guimarães

**Affiliations:** ^1^ Animal Science Graduate Program Federal University of Pará Castanhal Pará Brazil; ^2^ Biological Sciences Institute, Federal University of Pará Belém Pará Brazil; ^3^ Brazilian Agricultural Research Corporation (Embrapa) Eastern Amazon Belém Pará Brazil

**Keywords:** gonadal differentiation, gonadogenesis, *Kinosternon scorpioides*, ovary, testis

## Abstract

Research on gonadal development including sexual differentiation of gonads in chelonians is essential for understanding the mechanisms that determine offspring sex and, consequently, the sex ratio of egg clutches. This directly impacts population structure, as sex ratios influence reproductive dynamics and species' long‐term viability. The aim of this study was to characterize the gonadal development of scorpion mud‐turtle (*Kinosternon scorpioides*). A total of 85 embryos from stages 12–26 were analyzed, kept under digital temperature control (28.5 ± 0.5°C). The reproductive system was dissected and processed for histological analysis using light and transmission electron microscopy. The average incubation time was 117.35 ± 9.5 days. From stage 12 onwards, gonadal ridges containing primordial germ cells (PGCs) were observed. At stage 14, the undifferentiated gonads consisted of cuboidal coelomic epithelial cells that invaginated to form cord‐like structures containing germ cells. At stage 20, at approximately 50 days of incubation, sexual differentiation of gonads was noted. The ovaries showed a cortical region containing oogonia and oocytes, and a medullary region with a stroma rich in vessels. The testes were characterized by a peripheral band of dense connective tissue beneath the tunica albuginea, and by the presence of seminiferous tubules occupying the central portion of the organ. The results of this study provide important information on the gonadal development of *K. scorpioides*, as the identification of the moment of gonadal differentiation, stage 20, makes it possible to understand the influence of the environment on sex determination. These findings provide support for the reproductive management of *K. scorpioides* and can be applied directly to the sexual control of populations in situ and ex situ.

## Graphical abstract

Stage 20 was identified as the critical point for gonadal differentiation in *Kinosternon scorpioides*, providing key insights into sex determination. These findings enhance conservation strategies by supporting reproductive management and population viability in both in situ and ex situ programs.

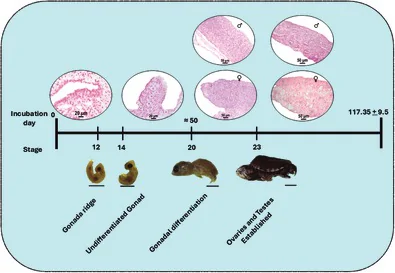

## 1 INTRODUCTION

The Amazon harbors one of the richest chelonian biodiversities in the world, with approximately 21 species identified (Rhodin, 2021). However, the intense capture of natural stocks, carried out without criteria and including animals of different life stages, sizes, and often without definition of sex (Costa et al., 2017), is an issue that deserves attention in terms of turtle species conservation. In this context, captive breeding has emerged as a viable alternative for obtaining and conserving these resources (Andrade et al., 2021; Garcez et al., 2021).

One of the main species of Amazonian chelonians, *Kinosternon scorpioides*, is also one of the most often consumed species on the northern Brazilian coast (Costa et al., 2017; Medeiros et al., 2023). This small, semi‐aquatic species inhabits a wide variety of aquatic environments, both permanent and temporary, lotic or lentic (Ferrara et al., 2017). In natural environments, this species lays eggs three to five times during the breeding season, in clutches of around four eggs (Ferrara et al., 2017). They reach sexual maturity at 2 years of age in captivity (de Castro, 2006), respond well to growth and weight gain inputs, and are easy to keep (Costa et al., 2017). However, despite the accumulated knowledge about habitat and reproductive characteristics, the process of gonad formation and differentiation in this species is still poorly understood.

Understanding the processes of gonadal differentiation and sex determination is essential for achieving progress in turtle farming and allowing improvements in the maintenance and management of breeding stock (Braga et al., 2024; Costa et al., 2017; Oliveira et al., 2021). To ensure successful reproduction in captivity, it is essential to understand the species' reproductive biology and hatchery management, including knowledge of the ideal conditions for gonadal development. These factors directly influence hatching rates, nestling production, and the sex ratio of captive populations (Braga et al., 2024).

In many chelonian species, sex determination is influenced by environmental factors, especially temperature (Ewert & Nelson, 1991; Ferreira Júnior, 2009). This process occurs during the thermosensitive period (TSP), which corresponds to the second third of the incubation period (Wibbels et al., 1998). Within a specific temperature range, the formation of both sexes occurs in equal proportions (50% males and 50% females, or a sex ratio of 1:1), this temperature being known as pivotal or central (Yntema & Mrosovsky, 1982). For sea turtles, the estimated pivotal temperature is approximately 29°C (Tanabe et al., 2020), while for the species *Podocnemis expansa* it is 33.5°C (Lubiana & Ferreira, 2009). In *K. scorpioides*, sex determination has been described as pattern II, with females developing at extreme temperatures and males at intermediate temperatures (Ewert & Nelson, 1991), and a pivotal temperature of 27.5°C has been reported (Ewert et al., 2004; Ewert & Nelson, 1991).

The gonads originate from the gonadal ridge, which develops as a thickening in the medial region of the mesonephric kidneys (Piprek et al., 2016). The ridge is lined by a coelomic epithelium formed by mesothelial cells (Wibbels et al., 1991) and is composed of mesenchymal cells and germ cells. In males, during embryonic development, the medullary cords of the primordial gonad differentiate into seminiferous tubules (Oliveira et al., 2021). In contrast, in females, the cells of the cortical epithelium and the germ cells proliferate, forming the primordial ovarian follicles (Magalhães, 2017).

The aim of this study was to describe the development and gonadal differentiation of *K. scorpioides*, based on the incubation of embryonated eggs under controlled temperature and humidity. Considering that gonadal differentiation occurs gradually and begins with the formation of specific structures, we hypothesize that during embryonic development of *K. scorpioides*, the initially undifferentiated gonadal cords arise early and subsequently follow sex‐specific pathways: in presumptive testes, these cords reorganize to give rise to the seminiferous tubules, whereas in presumptive ovaries, the medullary cords regress while the cortex becomes progressively enriched with germ cells, ultimately resulting in the distinct morphological characteristics of ovaries and testes.

## 2 MATERIALS AND METHODS

### 2.1 Study area

Specimens of *K. scorpioides* were obtained from the scientific breeding facility of the Brazilian Agricultural Research Corporation (Embrapa) (LO 7310/2014‐SEMAS‐PA), located on Marajó Island (Salvaterra, Pará, Brazil, 48°33′23.32″W and 0°42′25.26″S).

The breeding enclosure had a breeding area (80 m^2^), consisting of a water tank and a sand area where the eggs were laid. The adults (*n* = 138 females; *n* = 58 males), aged over 3 years, weighed an average of 500 g (Costa et al., 2017). The animals were given commercial feed (32% crude protein) three times a week, based on 1% of their body weight.

### 2.2 Artificial incubation

The eggs were incubated following the protocol described by Braga et al. (2024). Fertilized eggs were identified by candling and individually placed in plastic trays containing vermiculite. The trays were inserted into an Ecological Premium® IP‐R incubator, maintained at a controlled temperature of 28.5 ± 0.5°C and 80% relative humidity. The substrate was prepared using a kiln, according to Embrapa (1997), and dried at 95–110°C for 72 h. Humidity was monitored daily and kept constant at 80%. To maintain adequate moisture levels, the trays were weighed twice a week and water was added when necessary (Braga et al., 2024).

### 2.3 Embryo morphology

The eggs were collected from 19 nests, each containing one to five eggs. The embryos were euthanized using 0.5–2.0 mL of lidocaine hydrochloride (2%) (Magalhães et al., 2017). The entire procedure performed in this study was approved by the Animal Use Ethics Committees (no. 1459280520, meeting 07/07/2020) of the Federal University of Pará, Brazil.

The embryos (*n* = 85) were classified according to the morphological descriptions of the embryonic stages of *K. scorpioides* (Braga et al., 2021). Samples with a carapace‐plastron complex (from stage 17 onwards) were excised in the region of the bridge, then the plastron was removed, and a longitudinal incision was made from the mandibular region to the pelvis. All the pectoral and abdominal muscles were removed and the coelomic cavity was exposed for gonad removal.

The gonads of *K. scorpioides* embryos at different developmental stages were fixed in Bouin's solution for 24 h and processed using standard histological procedures for paraffin embedding. All samples were sectioned at 5 μm thickness using a Leica© microtome, stained with hematoxylin and eosin, and photographed under a light microscope (NIKON Eclipse Ci©) equipped with a digital camera (NIKON DS‐Ri1©). Images were edited using Adobe Photoshop© (Adobe Inc., San Jose, CA, USA).

Fragments of the gonads were also fixed in Karnovsky's solution (4% paraformaldehyde, 2% glutaraldehyde in 0.1 M sodium cacodylate buffer, pH 7.2) for 24 h (Karnovsky, 1965). They were then washed in 0.1 M sodium cacodylate buffer, pH 7.2, followed by post‐fixation in 1% osmium tetroxide in 0.1 M sodium cacodylate buffer, pH 7.2 for 2 h. The samples were dehydrated in an increasing series of acetone, infiltrated and embedded in Eppon 812. Ultra‐thin sections were made on an ultramicrotome, contrasted with uranyl acetate and lead citrate and analyzed on a CLARA high‐resolution SEM‐STEM (TESCAN, Brno, Czech Republic).

## 3 RESULTS

The average incubation time was 117.35 ± 9.5 days. The gonads were found attached to the ventromedial region of the kidneys (Figure 1a,b). The gonadal ridges of *K. scorpioides* embryos first appeared at stage 12, around 20 days of incubation. Sexual differentiation into testes and ovaries began later, during stage 20, at approximately 50 days of incubation. The chronology of gonadal development is summarized in Table 1.

**FIGURE 1:**
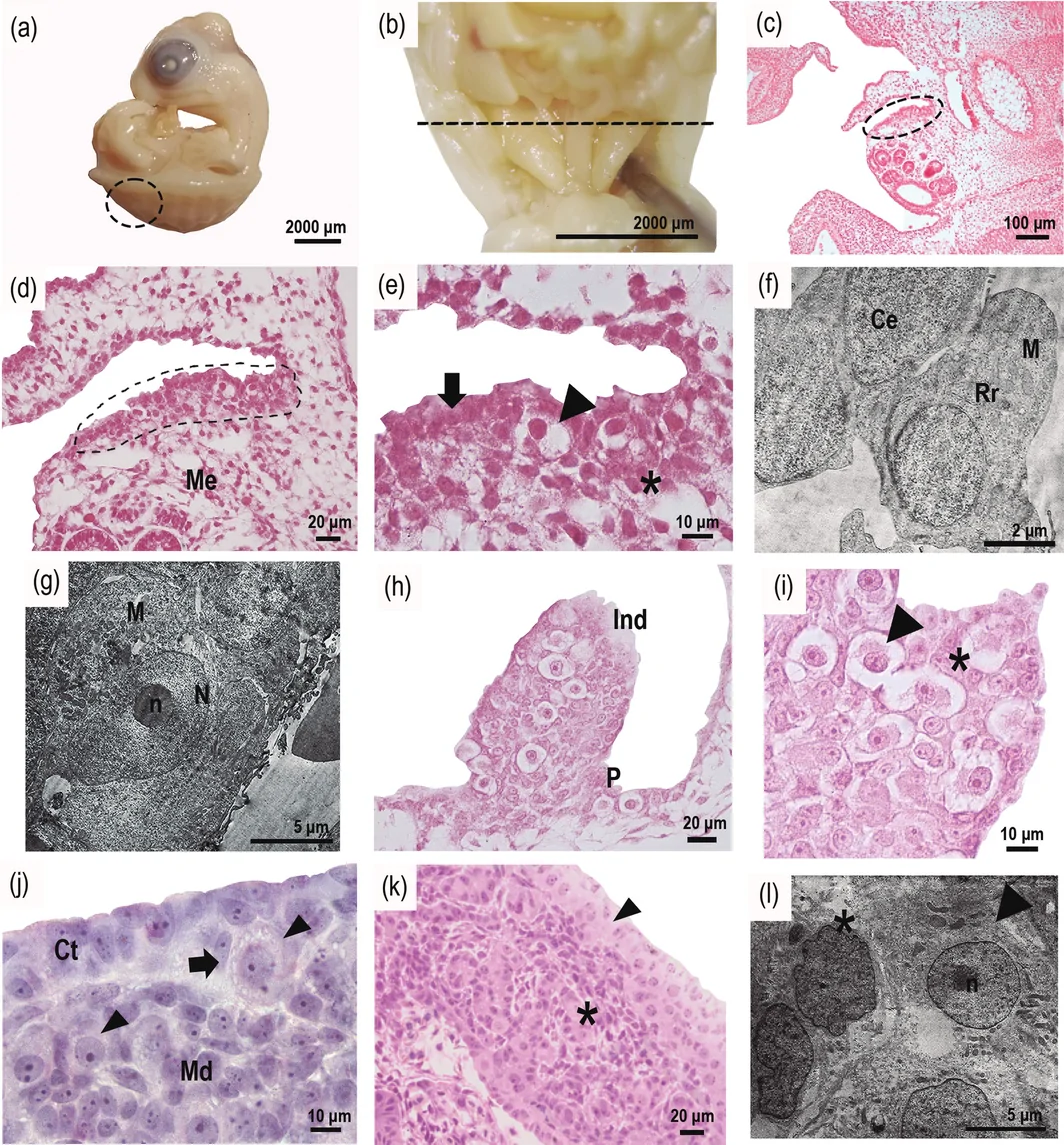
Photomicrograph of the gonad of *Kinosternon scorpioides* in undifferentiated stages. (a) *K. scorpioides* embryo showing the location of the urogenital system. (b) Macroscopic view of the urogenital system, with an inset showing the transverse section seen in (c), which highlights the position of the genital ridges. Stage 12: (d) Region of the gonadal ridge (dotted), underlying mesonephros (Me); (e) Coelomic epithelium (black arrow), germ cell (black arrowhead), mesenchymal cells (*). (f) Gonadal ridge, with the presence of coelomic epithelium cells (Ce), rough endoplasmic reticulum (Re) filled with mitochondria (M); (g) PGCs in the gonadal ridge, with a nucleolus (n) evident in the nucleus (N), filled with mitochondria (M), in the cytoplasm. Stage 14: (h) undifferentiated gonad (Ind), connected to the mesonephros by the peduncle (P); (i) Numerous germ cells (black arrowhead) arranged randomly in the gonadal epithelium, surrounded by cells that organize themselves to form the gonadal cords (*). Stage 16: (j) undifferentiated gonad where most of the germ cells (black arrowhead) can be seen in the cortical region (Ct), and few in the medullary region (Md), germ cell adjacent to germinal epithelium cell (black arrow). Stage 18: (k) cortical region, PGCs (black arrowhead) were unevenly distributed in several layers. In the medullary region, the gonadal cords (*) became more evident, and the presence of PGCs was rarely noticed. (l) gonadal cord showing germ cells (black arrowhead) with a large nucleus, dispersed chromatin and a prominent nucleolus (n), surrounded by mesenchymal cells (*).

**TABLE 1:** Embryonic stage correlated with the age (days) of embryos and the characteristic of gonadal differentiation (Und: Undifferentiated gonad; M: Male; F: Female).

*Kinosternon scorpioides* embryo's stage^a^	Aproximate age (days)	Sex (*n*)	Degree of gonadal differentiation
12	20	Und (3)	Early gonadal ridge identifiable
13	25	Und (3)	Early gonadal ridge identifiable
14	30	Und (4)	Gonadal ridges establishment
15	33	Und (4)	Gonadal ridges establishment
16	35	Und (3)	Gonadal ridges establishment
17	38	Und (3)	Gonadal ridges establishment
18	41	Und (5)	Gonadal ridges establishment
19	45	Und (4)	Gonadal ridges establishment
20	50	M (3) F(3)	Differentiating gonads
21	55	M (3) F (4)	Differentiating gonads
22	60	M (3) F (4)	Differentiating gonads
23	65	M (5) F (3)	Differentiating gonads
24	75	M (5) F (3)	Differentiating gonads
25	85	M (5) F (3)	Differentiating gonads
26	100	M (6) F (6)	Differentiating gonads

^a^ Braga et al. (2021).

### 3.1 Gonadal ridges

This study focused on gonadal development from stage 12 onward, when primordial germ cells (PGCs) were already localized within the urogenital crest. The migration process of PGCs to the urogenital crest was not evaluated.

During stage 12, the formation of the urogenital crest was noted (Figure 1c) and consisted of the thickening of the ventro‐medial region of the mesonephric kidneys (Figure 1d). The crest was made up of a coelomic epithelial lining and internally contained PGCs and mesenchymal cells (Figure 1e). Mesenchymal cells, located just beneath the coelomic epithelium, exhibited a thin cytoplasm and pale nuclei with dispersed chromatin (Figure 1e). The cells of the coelomic epithelium were cuboidal and contained rough endoplasmic reticulum adjacent to the nucleus and mitochondria (Figure 1f). The PGCs were bulky and rounded, with a large nucleus and dispersed chromatin, an obvious nucleolus, and pale cytoplasm with cytoplasmic organelles such as mitochondria (Figure 1g).

### 3.2 Undifferentiated gonad

At stage 14, the gonads were connected to the mesonephros by a peduncle composed of loose mesenchymal tissue covered by a single layer of coelomic epithelial cells, marking the onset of undifferentiated gonad formation (Figure 1h). The epithelium was composed of cuboidal cells and, as the epithelial cells invaginated, they formed cord‐like structures together with the germ cells (formerly PGCs) (Figure 1i).

In embryos from stage 15 onwards, the undifferentiated gonads were organized into two distinct regions: cortical and medullary. At stage 16, the presence of germ cells was evident in the cortical region, while in the medullary region, the first gonadal cords began to appear (Figure 1j).

From stage 18 onwards, both the cortical and medullary regions showed signs of greater definition and organization (Figure 1k). In the cortical region, the germ cells were unevenly distributed in several layers. In the medullary region, on the other hand, the gonadal cords became more evident (Figure 1k), and the presence of rounded germ cells with a large nucleus, dispersed chromatin, and a prominent nucleolus (Figure 1l) was rarely noticed.

### 3.3 Ovarian differentiation

From stage 20 onwards, at approximately 50 days of incubation, ovarian differentiation began. This process was marked by a progressive concentration of germ cells in the cortical region (Figure 2a) and the onset of structural reorganization in the medullary region (Figure 2b). At stage 21, the cortical region was densely populated with germ cells (Figure 2c), while the medullary region exhibited gaps associated with the expansion of proliferating blood vessels and a looser arrangement of stromal cells. These spaces, likely resulting from angiogenic activity and cellular rearrangement, became increasingly evident as the medulla developed. Gonadal cords were rarely observed at this stage, suggesting a low abundance.

**FIGURE 2:**
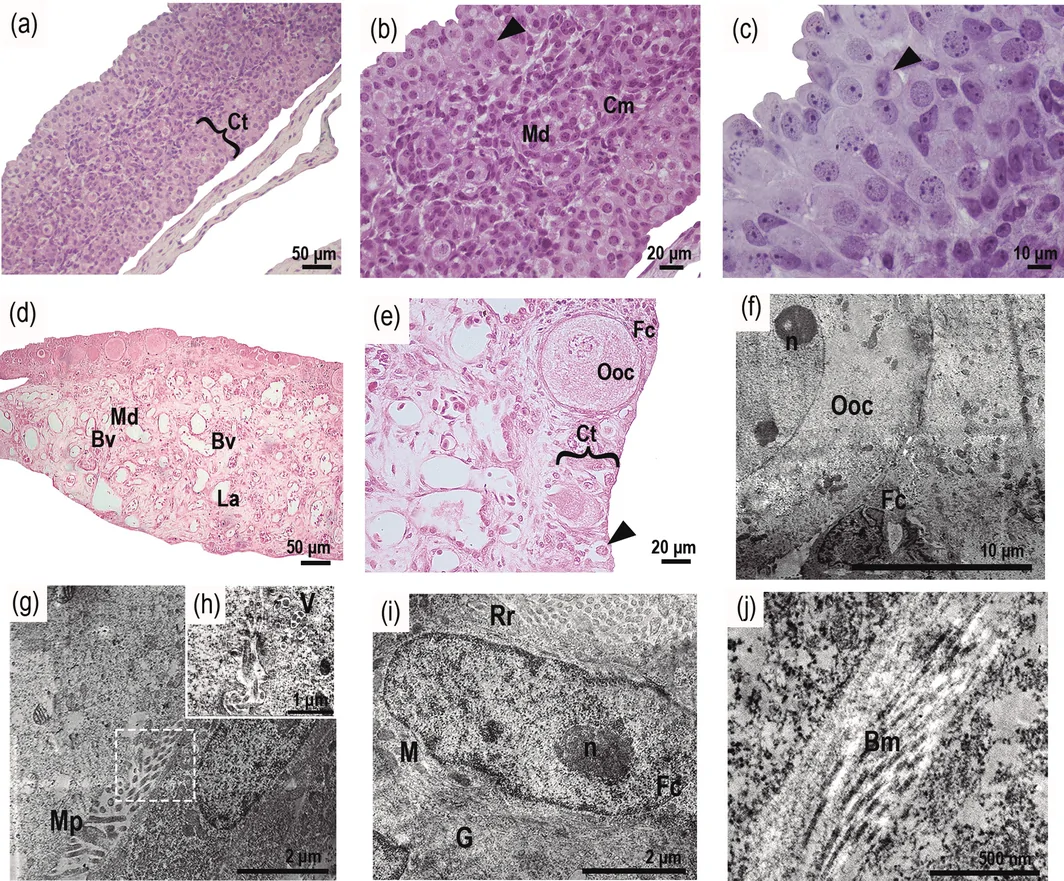
Photomicrograph of the ovarian differentiation of *Kinosternon scorpioides*. Stage 20: (a) beginning of ovarian differentiation, with germ cells in the cortical region (Ct). (b) Numerous layers of germ cells (black arrowhead) proliferating in the cortical region; in the medullary region (Md), gonadal cords (Cm) appear fragmented, indicating early signs of vascular proliferation and tissue reorganization. Stage 21: (c) layers of germ cells (arrowhead). Stage 26: (d) medullary region (Md) filled with blood vessels (Bv) and developing lacunae (La); (e) cortical region (Ct) containing oocytes (Ooc) surrounded by follicular cells (Fc) and by oogonia (black arrowhead). (f) Oocyte (Ooc) with evident nucleoli (n), partially surrounded by flattened follicle cells (Fc); (g) surface of the oocyte where numerous membrane projections (Mp) can be seen; (h) Highlight, where secretion vesicles (V) can be seen, being transferred to the oocyte; (i) evident nucleolus (n) of flattened cell (Fc), in the cytoplasm mitochondria (M), Golgi vesicles (G), and rough endoplasmic reticulum (Rr); (j) basement membrane (Bm) adjacent to the flattened cell on the surface of the oocyte.

At stage 26, the female gonads (Figure 2d) were identified by a lining epithelium composed of cuboidal cells and a cortex containing abundant oogonia, while in the medullary region, wide lacunae were observed among the blood vessels, likely corresponding to developing spaces within the medullary cords. The oogonia exhibited decondensed chromatin with some regions of condensation, and a follicular structure surrounded by flattened follicular cells was also identified, characterizing an ovarian follicle (Figure 2e).

Oocytes with prominent nucleoli were also observed at this stage (Figure 2f), in the cortical region near the medulla. Additionally, cytoplasmic projections and secretory vesicles were noted (Figure 2g,h). The oocytes were partially or completely surrounded by a layer of flattened (follicular) cells that contained a distinct nucleolus (Figure 2i). A thin basement membrane was observed surrounding the follicular cells, delimiting the ovarian follicles (Figure 2j).

### 3.4 Testicular differentiation

Testicular differentiation began at stage 20, approximately 50 days after incubation. At this stage, the lining epithelium consisted of cuboidal cells, and the gonadal cords were arranged in parallel structures extending from the periphery toward the medulla, with germ cells distributed predominantly in the cortical region (Figure 3a,b). At stage 21, germ cells (Figure 3c) were located within the gonadal cords, which began to exhibit a central lumen, indicating the initial formation of seminiferous tubules. These structures were surrounded by a thin basement membrane (Figure 3c). The interstitial tissue displayed loose connective tissue, composed of scattered mesenchymal cells and extracellular matrix. By stage 23, the epithelial lining had been replaced by a peripheral layer of dense connective tissue, identified by the increased presence of collagen fibers, consistent with the formation of the tunica albuginea.

**FIGURE 3:**
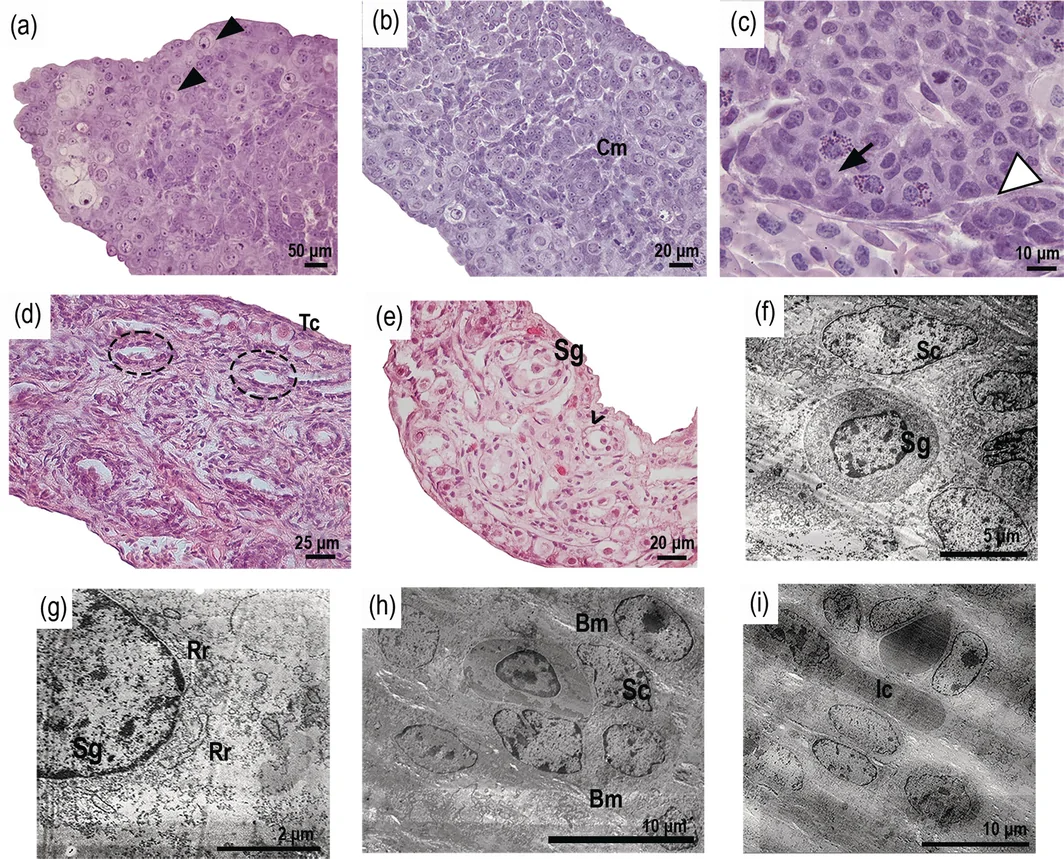
Photomicrograph of the gonad *of Kinosternon scorpioides* undergoing testicular differentiation. Stage 20: (a) Germ cells (black arrowhead) are seen in the cortical and medullary regions; (b) organization of the medullary cords (Cm). Stage 21: (c) Germ cells (black arrowhead) located within the testis cords, which show early lumen formation and are surrounded by a thin basement membrane (white arrowhead). Stage 26: (d) Seminiferous tubules with apparent lumen (dotted), and the tunica albuginea of dense connective tissue (Tc); (e) Spermatogonia (Sg) inside the tubule, and myoid cells (<). (f) Seminiferous tubule with detail of Sertoli cell (Sc), adjacent to a spherical‐shaped spermatogonia (Sg); (g) Spermatogonia (Sg), note the rough endoplasmic reticulum (Rr) adjacent to the nucleus. (h) Seminiferous tubule, Sertoli cell (Sc), surrounded by basement membrane (Bm). (i) myoid cell (Ic) with condensed chromatin.

At stage 26, the testicles presented seminiferous tubules (Figure 3d–f) with a clear lumen. These tubules were lined by large, round‐shaped cells, identified as spermatogonia, which exhibited a central nucleus containing dispersed chromatin with some regions of condensation. Ultrastructural analysis showed that these cells contained abundant rough endoplasmic reticulum in their cytoplasm (Figure 3g). Elongated Sertoli cells, with basal nuclei, surrounded the spermatogonia (Figure 3h). Lining each seminiferous tubule were flattened myoid cells, with condensed chromatin (Figure 3i) and a surrounding layer of loose connective tissue composed of collagen fibers.

## 4 DISCUSSION

The results of this study showed that in *K. scorpioides* eggs incubated at 28.5°C, it was possible to observe gonadal development from stage 12 onward, including gonadal differentiation. Gonadal differentiation occurred at stage 20 of embryonic development, approximately 50 days after the start of incubation. In Kinosternidae the sex determination depends on the incubation temperature of the eggs (Ewert et al., 1994), occurring during the TSP. This critical moment is recognized as coinciding with the second third of embryonic development in Testudines (Bull & Vogt, 1981; Wibbels et al., 1998). Based on this premise, studies indicate that, in *K. scorpioides*, the TSP should possibly cover stages 18–24 of embryonic development (Braga et al., 2021). To corroborate this hypothesis, experiments exploring different incubation temperatures are needed to accurately determine the TSP, both for the species analyzed here and for others in the Kinosternidae group.

It is important to note that the TSP varies between species, which highlights the uniqueness of each one in the context of gonadal development. In *Malayemis macrocephala*, for example, the TSP was identified between stages 13 and 17 (Pewphong et al., 2021), while in the yellow‐spotted Amazon river turtle (*Podocnemis unifilis*), it occurs between stages 11 and 19 (Souza & Vogt, 1994). This variation reinforces the fact that the TSP is specific to each species and reflects the evolutionary and environmental adaptations associated with embryonic development (Magalhães, 2017).

In this study, gonadal development in *K. scorpioides* was described in three stages: gonadal ridge, undifferentiated gonad and gonadal differentiation. These stages of development were also observed in the following species: (1) turtles—*Sternotherus odoratus* (Kinosternidae) (Risley, 1933), *Lepidochelys olivaceae* (Merchant‐Larios et al., 1989), *P. expansa* (Hildebrand et al., 1997; Magalhães, 2017), *Mauremys reevesii* (Akashi et al., 2022); (2) crocodilians—*Alligator mississippiensis* (Ferguson, 1985); (3) birds—*Gallus gallus domesticus*, *Anas platyrhynchos f. domestica* and *Columba livia domestica* (Mizia et al., 2023); (4) lizards—*Correlophus ciliatus, Eublepharis macularius*, and *Lepidodactylus lugubris* (Rams‐Pociecha et al., 2024). These findings highlight the fact that gonadal development in *K. scorpioides* and other vertebrates adheres to an evolutionarily conserved pattern. This suggests that similar mechanisms may have played a role in gonadal formation and differentiation throughout the evolutionary history of these groups (Bull, 1980; Ewert & Nelson, 1991; Vining et al., 2021).

The morphological changes observed during gonadogenesis in *K. scorpioides* were similar to those described in other reptiles, with development beginning from the thickening of the coelomic epithelium in the ventromedial region of the mesonephric kidney (Akashi et al., 2022; Oliveira et al., 2021). In this species, the gonadal ridge was already identifiable at stage 12, which coincides with the emergence of limb buds—specifically, the initial protrusion of the forelimb buds (Braga et al., 2021). This pattern is consistent with what has been described in other amniotes, such as lizards of the Gekkonidae family, birds, and amphibians, where gonadal ridge formation occurs in parallel with early limb bud development (Mizia et al., 2023; Piprek et al., 2017; Rams‐Pociecha et al., 2024).

In *K. scorpioides*, sexual differentiation of the gonads begins at stage 20, which corresponds to the morphological appearance of digital plates in both forelimbs and hindlimbs (Braga et al., 2021), similarly to what has been observed in other chelonians. This temporal overlap suggests a coordinated developmental timing between limb and gonadal morphogenesis. It is hypothesized that this synchrony may result from the shared activation of conserved signaling pathways, such as Sonic Hedgehog (Shh), known to be crucial for both limb outgrowth and the patterning of gonadal structures (Dilower et al., 2023; Tickle & Towers, 2017). Therefore, further comparative studies are warranted to investigate the genetic and molecular basis of this developmental coordination across vertebrates.

In vertebrates, PGCs migrate toward the gonadal region and complete their migration as the gonadal ridges begin to form (Piprek et al., 2016). These have a spherical shape and abundant cytoplasm (Oliveira et al., 2021; Wibbels et al., 1991). Glycogen particles and cell organelles such as the Golgi complex, rough endoplasmic reticulum, mitochondria, and free ribosomes have been identified in the cytoplasm of PGCs of the turtle *Caretta caretta* (Fujimoto et al., 1979). The presence of these cellular components highlights the fact that, during gonadogenesis, the cells of the gonadal ridge must be adequately prepared for the development and differentiation of this tissue (Fujimoto et al., 1979). In *K. scorpioides*, the PGCs of the gonadal ridge showed a characteristic arrangement in the protein synthesis function, and this aspect is involved in cell maturation during gametogenesis.

The undifferentiated gonad of *K. scorpioides*, at stage 14, showed expansion of the stroma and inclusion of the germ cells. As observed in this study, the undifferentiated gonad contained a cortical region with germ cells and a medullary region with gonadal cords, similar to that found in other chelonians (Pieau et al., 1999). In the species *S. odoratus*, the migration of PGCs to the gonad also contributed to tissue development (Risley, 1933). With the establishment of the undifferentiated gonad, the gonadal cords proliferate in the medullary region, and it is known that these are formed by the proliferation of the germinal epithelium (Pieau et al., 1999). These processes are essential for the initial formation of the undifferentiated gonad, which forms the basis for future differentiation into testes or ovaries and highlights the complexity and relevance of gonadal development in chelonians.

In this study, gonad differentiation occurred from stage 20, at approximately 50 days of incubation. In the turtle *P. expansa* it was observed from the 35th day of incubation, at stage 19 (Hildebrand et al., 1997; Magalhães, 2017). In the turtle species *M. macrocephala* it occurred at stage 17 in males, and 19 in females (Pewphong et al., 2020). In other vertebrates, sexual differentiation of gonads, although regulated by sex chromosomes, occurs concomitantly with the curling of the edge of the digital plate of the limbs, which gives rise to the digits (Mizia et al., 2023; Rams‐Pociecha et al., 2024). In *K. scorpioides*, at stage 18, when the digits appear (Braga et al., 2020), the gonad is divided into cortical and medullary regions, although it remains undifferentiated. Although there are similar developmental patterns between species, the complexity of the sexual differentiation of gonads process can vary, which reflects specific evolutionary adaptations in response to different factors.

These interspecific differences in the timing of gonadal differentiation may be influenced by multiple factors, such as incubation temperature, embryonic growth rate, metabolic rate, and the timing of hormonal signaling involved in sex determination (Singh et al., 2020). Species with temperature‐dependent sex determination (TSD), for example, often exhibit shifts in the onset of differentiation depending on thermal regimes (Gammon et al., 2020; Valenzuela & Lance, 2004; Wibbels et al., 1991). In addition, ecological pressures such as nest depth, climate variability, and reproductive seasonality may select for variations in developmental pace and differentiation timing (Du et al., 2019). Thus, the diversity in gonadal development observed among chelonians likely reflects not only phylogenetic divergence but also adaptive responses to species‐specific reproductive and environmental contexts.

During ovarian differentiation in *K. scorpioides*, two processes accompany the development of the ovary. The cortex persists, housing the oogonia, and the gonadal cords regress into the medulla. The *P. expansa* turtle shows invaginations in the gonadal surface epithelium, which remain after ovarian differentiation, giving the ovary a pleated appearance (Hildebrand et al., 1997; Magalhães, 2017). In contrast, this characteristic was not observed in the gonads of *K. scorpioides*. The thickening of the cortex occurs due to the proliferation of the PGCs, which formed the clusters of oogonia; this cell proliferation was clear from stage 18 onwards and was maintained prior to hatching. At stage 26, in addition to the oogonia, follicles with flattened cells were observed, surrounding the *K. scorpioides* oocytes. However, this conformation has not been observed in the pre‐hatching stage of *L. olivaceae and M. reevesii* turtles (Akashi et al., 2022; Merchant‐Larios et al., 1989).

In mammals such as mice, follicular cells and Sertoli cells have been shown to originate from the coelomic epithelium during gonadal differentiation (Liu et al., 2016). In contrast, in birds like chickens, supporting cells have been reported to derive from the mesenchyme (Estermann et al., 2020). In *Trachemys scripta* and geckos, medullary cords appear to emerge as extensions of the cortical region, suggesting partial cortical contribution to medullary formation (Rams‐Pociecha et al., 2024; Yao et al., 2004). According to Guraya (1989), the process of gonadal organization in reptiles appears to differ from that observed in mammals and birds, but the specific origin of supporting cells (Sertoli and follicular cells) remains unclear. In *K. scorpioides* and other reptiles, the origin of these supporting cells remains unclear, and further studies are needed to better understand the postnatal development of oogonia and follicular cells.

During ovarian differentiation there was regression of the gonadal cords in the medulla, which fragmented, spaced out, and formed a vascular stroma similar to that found in *S. odoratus* (Risley, 1933). In contrast, in *C. caretta* the gonadal cords persist (Yntema & Mrosovsky, 1980); this retention has also been observed in the *Apalone spinifera* turtle (Greenbaum & Carr, 2001). These patterns indicate that the organization of the ovarian medulla may vary significantly between chelonian groups, reflecting differences in the regulation of gonad development and gonadal differentiation.

During testicular differentiation, two processes were involved: the growth and differentiation of the seminiferous cords into seminiferous tubules, and the reduction of the cortical region (Oliveira et al., 2021). In *P. expansa*, the surface epithelium was reduced, but a layer of cells was observed covering the gonad (Hildebrand et al., 1997; Magalhães, 2017). In contrast, in *K. scorpioides*, the lining epithelium became thinner, while a connective tissue tunic, the tunica albuginea, developed beneath the epithelium prior to hatching. These specific variations in the lining of the organ may reflect reproductive adaptations linked to the physiological needs of each species.

In some species, sexual differentiation of gonads does not occur synchronously between the male and female sexes. In *L. olivaceae*, ovaries were observed developing earlier than the testes (Merchant‐Larios et al., 1989). This variation also occurs in other reptiles, for example, in *A. mississippiensis* the testis differentiates earlier than the ovaries (Smith & Joss, 1993). However, in *K. scorpioides*, during stage 20, the ovaries and testes differentiate, as in the turtle *Trachemys scripta* (Wibbels et al., 1991).

It is essential to consider the specificities of each species with regard to the timing of gonadal differentiation, especially in relation to the thermosensitive period, in order to optimize incubation practices and aid breeding programs. The data from this study highlight the complexity and variations in the processes of gonadal development in *K. scorpioides*, showing how intrinsic and environmental factors, as well as incubation temperature, can influence sex determination and reproductive success. Although there are conserved patterns among vertebrates, the unique evolutionary adaptations of each species highlight the importance of deepening our understanding of the events of gonadogenesis. This knowledge not only enriches the understanding of the reproductive biology of chelonians but also supports effective strategies for conservation, nest management, and the protection of endangered populations.

## AUTHOR CONTRIBUTIONS

**Brenda Braga:** Data curation; formal analysis; writing – original draft; methodology; conceptualization. **Verônica Oliveira‐Bahia:** Conceptualization; writing – original draft; writing – review and editing. **Maria Auxiliadora Ferreira:** Resources; writing – original draft; writing – review and editing. **José Ribamar Felipe Marques:** Resources. **Diva Araújo Guimarães:** Funding acquisition; conceptualization; writing – original draft; writing – review and editing.

## CONFLICT OF INTEREST STATEMENT

The authors declare that they have no known competing financial interests or personal relationships that could have appeared to influence the study reported in this paper.

## Data Availability

The datasets generated during and/or analyzed during the current study are available from the corresponding author on reasonable request.
